# Tofersen and other antisense oligonucleotides in ALS

**DOI:** 10.1177/17562864251313915

**Published:** 2025-01-22

**Authors:** Albert Ludolph, Maximilian Wiesenfarth

**Affiliations:** Department of Neurology, Ulm University, Oberer Eselsberg 45, Ulm 89081, Germany; Deutsches Zentrum für Neurodegenerative Erkrankungen, Ulm, Germany; Department of Neurology, Ulm University, Ulm, Germany

**Keywords:** amyotrophic lateral sclerosis, antisense oligonucleotides, superoxide dismutase, tofersen

## Abstract

The advent of antisense oligonucleotide (ASO) therapies in neurodegenerative disorders is associated with enormous hope. Nusinersen treatment was a breakthrough intervention in the recessive disease spinal muscular atrophy, and superoxide dismutase 1 (SOD1) amyotrophic lateral sclerosis (ALS) seems to be the paradigm disease in dominant degenerative diseases. The results of treatment with the ASO tofersen in SOD1-ALS show that the drug has a convincing beneficial effect on ALS caused by SOD1 mutations, that preclinical studies in rodents predicted the therapeutic effect in the human disease, and that clinical efficacy is associated with a specific sequence of effects of the drug on mechanistic and degenerative biomarkers and, subsequently, functional outcomes such as weight stabilization and ALSFRS-R. Therefore, the enthusiasm seems to be justified; but this should be followed by an attempt to obtain further insights with the goal to improve this therapy. In particular, the following issues are only partially resolved: Which mechanisms are responsible for the clinical effect following the downregulation of SOD1 protein by ASOs? Is long-term downregulation of SOD1 function associated with side effects? Is there an autoimmune response caused by this and other ASO? Is prevention of SOD1-associated ALS possible?

## Familial and sporadic ALS

It is estimated that familial amyotrophic lateral sclerosis (ALS) accounts for about 5% of ALS cases.^1,2^ In line with these findings, 5% of ALS patients in an established register in Germany reported a family history of the disease.^3^ In addition, it was shown that a number of ALS-associated mutations are also observed in seemingly sporadic patients; this is true for superoxide dismutase 1 (SOD1),^4^ for fused in sarcoma (FUS),^5^ but particularly for chromosome 9 open reading frame 72 (C9ORF72) patients which seem to lack a family history in about 50% of cases in Central Europe.^6,7^ The number and the spectrum of ALS-associated mutations differ around the world in heterogenous ethnicities; variants and their impact must therefore be seen against a specific genetic background. This is true for China,^8,9^ Mongolia^10^ and particularly for populations with a comparatively high rate of consanguinity, such as Egypt.^11,12^ The pathogenicity of the individual genetic variants requires a detailed discussion.^7^

## Superoxide dismutase 1-amyotrophic lateral sclerosis

In 1993, Rosen et al.^13^ convincingly showed that mutations in the SOD1 gene cause ALS. In the meantime, numerous mutations were shown to be associated with the disease, but the relation of genotypical variation to the human phenotype is still not always defined.^7,14^ The mechanism by which the mutation leads to tissue damage has also not been clarified in a detailed manner. In contrast, there is a great deal of evidence that the formal pathogenesis of SOD1-associated ALS differs from the majority of ALS patients. Interestingly, while ALS is associated with the marker phosphorylated TAR DNA-binding protein (pTDP-43)^15^ in more than 95% of patients, SOD1-ALS can be modeled by a prion-like experimental mechanism.^16^ In Germany, 13% of familial ALS cases are associated with mutations in SOD1; in other parts of the world these numbers differ considerably.^9,12^ Recently, it has been shown that about 13% of 2267 seemingly sporadic patients in Central Europe carry a pathogenic ALS variant, among them around 40 patients with 35 SOD1 mutations^7^ which are associated with a lot of phenotypic variability, including clinical diseases with slower and faster progression.

The mechanism of resulting tissue damage in ALS has been explored by the use of rodent models, preferentially the G93A mouse which develops the motor phenotype within 4–5 months.^17,18^ Studies of this model showed that a toxic gain of function of the mutated gene is very likely to contribute to the phenotype of motor neuron dysfunction and damage. However, direct translation of therapeutic results obtained in G93A mice to humans is often met with failure. In many of the mouse models, however, the “dosage” of the overexpressed inserted genes was much larger than what has been observed in human patients; therefore, some of these failures of translation are thought to be due to overexpression artifacts. It is currently—after 25 years—still a matter of discussion how much the SOD1 models contribute to a mechanistic understanding of sporadic human ALS and which mechanisms described in the model are important for human disease and which could be rather considered to be secondary to overexpression.

## Animal studies

Attempts to influence the natural progression of SOD1-associated ALS were performed in the SOD1 mouse and rat models by lowering mutant SOD1 protein.^19^ The drug tofersen (BIIB067) served as a pharmacological tool; after intrathecal administration, it reduces the expression of the mutant protein by mediating RNase H-dependent degradation of the mRNA of SOD1.^19^ In experimental rodents, intrathecal administration of this ASO targeting SOD1 mRNA transcripts was safe and had a therapeutic effect on muscle loss, increased survival in these models, and lowered the axonal serum marker phosphorylated neurofilament heavy chain (pNfH). The therapeutic effect even seemed to include reversal of some disease markers, in particular the amplitude of the compound muscle action potential, indicating the presence of partial recovery in rodents. After intrathecal administration to cynomolgus monkeys the ASO was also shown, as in the rodent models, to dose-dependently reduce the SOD1 mRNA and protein concentrations in the CNS by about 50%; this decrease was mirrored by a decrease in CSF levels.^19^

Given the success of the human trials, the predictive efficacy of the ASO in the rodent models for human translation is of interest. In general, the translation of therapeutic effects in rodent models was previously not always successful; however, the important differences between the experimental tofersen studies and previous studies are

(a) That it was known that the mutated SOD1 is an important etiological factor for both the human and the rodent disease.
(b) For the first time, markers were used that monitor the effects of various dosages and the pharmacodynamics of the drug (mechanistic marker, SOD1 protein) and degenerative process (degenerative marker, pNfH).

## Results of the tofersen trials in humans

The design of the tofersen phases I–II and II–III trials aiming to translate the animal results to humans was innovative. On the one hand, the established El Escorial criteria were not used for recruitment, rather the 1 + 1 rule as suggested by the WFN group on ALS was used for early diagnosis for the first time.^20,21^ To improve early inclusion into trials, these criteria for the diagnosis of ALS only request the presence of both upper and lower motor neuron signs at a single limb. Alternatively, the 1 + 1 rule is fulfilled if two limbs are affected by the disease. It was important for the inclusion criteria of the tofersen studies that, in the case of genetic diseases, the 1 + 1 rule is also fulfilled if a lower neuron deficit is seen clinically at a single extremity and—in addition—the patient carries a disease-causing mutation.^7,21^ This criterion was the basis of early inclusion of patients carrying a SOD1 mutation into the therapeutic trial.

The second important innovation was the use of mechanistic and degenerative biomarkers as outcome measures in addition to functional clinical endpoints; as in animals, the effect of the drug—and its dosage—was demonstrated and controlled by the measurement of SOD1 protein expression (“mechanistic biomarker”) in CSF. In addition, the effect on degeneration was quantified by using the neuroaxonal marker neurofilament light chain (NfL) in blood. This marker has been shown to mirror the severity and progression rate of the disease,^22,23^ and its use is complementary to the neuroanatomical concept of ALS as a primary disease of corticoefferent axons as established by Braak et al.^15^

In the first phase I–II tofersen trial,^24^ it was shown that multiple intrathecal dosing of the drug up to 100 mg induced only minor side effects; most were related to lumbar puncture such as reversible headache and local pain. A dose-dependent increase of plasma concentrations of the drug could be observed, and it was found that tofersen crossed the blood–brain barrier and its concentration in the CSF increased nonlinearly. The SOD1 protein levels were decreased in CSF with a maximum reduction of about 35% after the administration of the 100 mg dosage at day 85. During this observation period, reductions in NfL and pNfH in plasma and cerebrospinal fluid were also reported in the same subgroup. An exploratory interim analysis after 3 months showed a tendency of stabilization of clinical measures such as motor function (measured by the ALSFRS-R), respiratory function (% predicted vital capacity, SVC), and muscle strength (hand-held-dynamometry, HHD megascore), when compared to controls. Since these 3-months pilot results were promising, the conduction of a phase II–III trials (VALOR) was the next step.

For the VALOR phase II–III study—a placebo-controlled, prospective, double-blind phase II–III trial—two cohorts representing fast and slow progression of the disease were studied. These two cohorts could be readily distinguished by the rate of decline of the ALSFRS-R as well as the NfL levels.^25^ The results of the intervention did not show major differences in both groups.

At first sight, the results of the phase II–III trials were disappointing.^25^ The study statistically significantly reproduced the effects of tofersen on mechanistic (target engagement) and degenerative biomarkers (axonal damage), but only showed a positive tendency of an effect on functional motor scores. Disappointingly, this effect was not statistically significant after 6 months.

Retrospectively, the first positive clinical effect was an anticatabolic effect, meaning that the application of the drug stabilized body weight after 6 months^26^; however, this outcome measure was not part of the intention to treat analysis. After another 6 months of an open label extension (OLE) study (overall 52 weeks), the effect on the motor score (ALSFRS-R) crossed the level of significance (*p* = 0.0272). The same was true for the secondary functional endpoints SVC and HHD (*p* = 0.0186 and *p* = 0.0159, respectively). Importantly, in this OLE phase, the noninterventional (placebo) group also stabilized under drug treatment, and an effect on mechanistic and functional biomarkers was shown.^26^

In the meantime, the Food and Drug Administration accepted the major effect of the drug on the axonal marker NfL as an evidence for the effectiveness of tofersen and approved the drug for the United States in spring 2023; the European Medicines Agency followed after 1 year.

In the meantime, an observational cohort study of the long-term effectiveness in the real world, in the framework of the Early Access Program in the German ALS/MND group, was published.^27^ In principle, after 12 months, the findings of the VALOR study were reproduced. In these 24 patients, the group—beyond VALOR—could show that both axonal markers, pNfH in CSF and NfL in serum, convincingly decreased (*p* = 0.02) after 3–6 months and then stabilized on a low level. The decline of the ALSFRS-R (“progression rate”) slowed down (*p* = 0.04). In some patients, an autoimmune response was observed in the CSF, but this was only accompanied by clinical symptoms in one patient who was diagnosed with autoimmune myeloradiculitis. However, tofersen treatment could be continued only after symptoms of myeloradiculitis were completely reversed due to immunomodulatory treatment. None of the patients died during the observation period.

Meyer et al.^28^ reported a positive effect of tofersen on NfL in serum in 6 German patients (partially overlapping with the first group) of the same network, and in a second paper, in 16 patients, showed also an effect on progression rate and NfL. The effects of treatment ranged from slowing of deterioration to even improvement.^29^ Therefore, by slowing down the progression rate in patients with SOD1-ALS, Saini and Chawla logically concluded in a comprehensive review of the literature that tofersen has the potential to break barriers in ALS therapy.^30^

## Other ASO trials in ALS

The success of the VALOR study and its OLE raised hopes that tofersen was not the only ASO to stabilize genetic subforms of ALS. Studies of antisense oligonucleotides (ASO) against other ALS-causing mutations followed: The C9ORF72 mutation, the most frequent mutation in the Western world (but not so in parts of Asia), was chosen as a next drug target. The FUS mutation which is prevalent all over the world and the disease modifier Ataxin-2 (ATXN2) were also seen as interesting targets.

Two ASO studies against the most frequent genetic cause of ALS in the Western world, C9ORF72, were conducted. The first randomized, placebo-controlled, dose-escalating phase I trial and the clinical development program by Biogen were discontinued in March 2022 as the investigational drug BIIB078 at a dose of 60 mg did not show a clinical effect, although the drug was well tolerated.^31^ A dosage of 90 mg seemed to induce a greater decline of clinical function in patients than placebo. The authors concluded that there must be many complex mechanisms, beyond toxicity, which are responsible for tissue damage by the C9ORF72 mutation. An improved understanding of the pathogenetic impact of these diverse mechanisms should be the basis of further drug development. The second study, performed by Wave Life Sciences, was discontinued in May 2023^32^ with the statement “that treatment with WVE-004 did not result in clinical benefit after 24 weeks.” The company commented that target engagement seemed to be reached since the poly(GP) biomarker reflecting the C9ORF72 hexanucleotide repeat expansion (G_4_C_2_) transcripts were lowered by 51% at week 24. However, this was not associated with a clinical benefit. Also, in an exploratory analysis, no effect on NfL was observed.

ALS caused by mutations in the FUS gene often has an aggressive course and frequently affects young patients. There is evidence that a nonallele-specific ASO silencing FUS and reducing FUS protein in the brain delays motor neuron degeneration in mutant FUS mice.^33^ After the intrathecal application of this ASO to a single patient, wild-type and mutant FUS levels were also lowered^33^; this justifies the hopes that the current ASO phase III trial (Ulefnersen, formerly ION363) run by IONIS might have chances of success. The trial is conducted as a multicenter, three-part study of ION363 in up to 95 participants (ClinicalTrials.gov ID NCT04768972); results are expected in late 2025.

There is experimental evidence that ATXN2 is a modifier of ALS; increased numbers of repeats lead to a more severe clinical picture. Therefore, reduction of the expression of the ATXN2 protein could also have a beneficial effect on the natural history of ALS. Unfortunately, in May 2024 the development of the drug BIIB105 had to be discontinued^34^ since—although the ASO reduced the ATXN2 protein levels in CSF—there was no effect on NfL levels after a 6 month placebo-controlled study. No effect on clinical outcome measures such as ALSFRS-R could be demonstrated. In addition, the open-label extension program did not yield any positive efficacy data.

## Tofersen as a role model

Tofersen is a successful interventional drug in SOD1-ALS. However, this is not the only message of the tofersen studies. Important additional aspects of the development of tofersen include that

**Figure 1.**
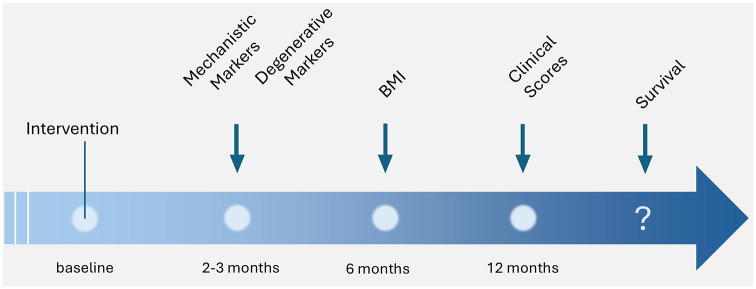
Temporal sequence of the effects of tofersen. The ASO was shown at first to reduce the amount of mutant—potentially toxic—protein, followed by levels of the axonal biomarker. Next, the body weight normalized (an outcome measure which was not in the intention-to-treat analysis), the ALSFRS-R, the HHD, and the SVC reacted to the treatment after more than 6 months. Survival data are not known. ASO, antisense oligonucleotide; HHD, hand-held-dynamometry; SVC, % predicted vital capacity.

(a) In contrast to previous studies in experimental animals, the rodent studies predicted the human response. This is most likely due to the fact that an approach to translation has been used which is likely to serve as a role model. The ambiguities of the use of anatomically and phylogenetically very different experimental species (such as mice) for drug development to a disease in which human-specific anatomical structure are affected^15^ have been reduced by defining the target (measurement of mutant protein, SOD1). This makes sure that the target has been hit and also reduces the ambiguities of interspecies dose-finding.
(b) In contrast with previous studies, relevant outcome measures for ALS—the axonal markers NfL and pNfH—have been shown to be altered by the intervention. Of note, it has only been shown by neuroanatomical studies that lesions of corticoefferent axons are a principle early component of the disease in the majority of TDP-43 associated ALS cases. It is not known yet whether SOD1-related ALS is associated with the same pathogenetic principle. However, the use of axonal biomarkers was shown to work in SOD1-ALS also and reduced the ambiguities of translation.
(c) Interestingly, it could be shown that a specific sequence of events of reactions to the interventions could be observed: at first (after weeks to months) the reaction of the target (or the mechanistic marker) SOD protein and the degenerative marker (NfL, pNfH) was influenced by the intervention. The effects on function (functional score ALSFRS-R) followed after 6–12 months, and the last endpoint which should be observed—survival—must follow (Figure 1).
(d) The clinical outcome measure of catabolism (or treatment of catabolism) was also influenced surprisingly early. Catabolism is an important clinical feature of ALS^35^; body weight stabilization was observed before a significant effect on the ALSFRS-R occurred, after only 6 months.^26^

In our view, the translational experience associated with the development of tofersen is a potentially intriguing lead into future interventions for diseases of the nervous system beyond ALS. The failure of the ASO trials against C9ORF72 and ATXN2 demonstrate that the combination of endpoints (mechanistic, neurodegenerative, clinical), rather than a single measure, should be preferentially used for successful translation.

## Open questions

### Is prevention of ALS possible?

After the successful studies of tofersen in experimental animals and humans, the idea of prevention of ALS comes into play. However, as long as the pathogenicity of a given mutation,^7^ its penetrance, the time of onset, and severity of the disease cannot be reliably predicted, preventive treatment is difficult. The step that is currently done worldwide in spinal muscular atrophy—the establishment of new-born-genetic-screening—can only be used with major caveats in ALS. If we do not know when and if a disease process begins, potentially as a result of low penetrance, then the balance between effects and side effects of even a successful drug becomes unpredictable for the individual patient. Therefore, for prevention we need early markers which reliably predict the onset of disease. How can this be achieved?

(a) A minimum prerequisite for early treatment is early clinical diagnosis. It is now widely accepted that the El Escorial criteria are not sufficient for early treatment and should be replaced by criteria using modern differential diagnostic tools.^20,21,36^ Although the suggested criteria (“1 + 1,” “Gold Coast”) are sensitive, the specificity of these diagnostic criteria must be validated; in addition, in a global world the phenotypical differences of ALS in different populations and ethnicities must be taken into account.^37^
(b) Preclinical gene carriers are a population in which new markers can be developed and in a second step be transferred to the population level. The first steps have been done.^38–40^
(c) Additional suggestions have been made for the definition of preclinical ALS, but there is a need for these ideas to be validated internationally in a collaborative, prospective study.^41^
(d) It is a major hurdle that in contrast to Parkinson’s and Alzheimer’s diseases, in ALS no preclinical period has been defined by anatomical methods.^42^ The first alterations are observed in the motor cortex and in the corticoefferent axons of ALS patients.^15^ Whether the hypothetical preclinical changes have an anatomical substrate is unknown.^35,41^

The ATLAS trial (by Biogen) takes all of this into account; it incorporates a scientific strategy by using NfL as a preclinical marker and a less risky plan B based on the development of early clinical deficits as defined by innovative diagnostic criteria.

### Autoimmune responses

In the first tofersen studies, a number of—in most cases—reversible immune reactions were observed.^25,27,43^ This includes reversible transverse myelitis and radiculitis. It will be of interest to further define, treat, and prevent these obvious autoimmune reactions. It might be therapeutically relevant to know whether the autoimmune response is specific for the interventional drug tofersen or whether it reflects nonspecific mechanisms.

### Long-term downregulation of SOD1 function

SOD1 is an important enzyme with antioxidative functions which are thought to be vital. The tofersen trials have shown that downregulation of the protein by roughly a third had no disadvantages for the patient during the time of observation. The question remains whether measurements of protein levels are sufficient for safety, additionally enzyme activity could also be considered. In addition, given the important functions of SOD1, it will be important to determine whether long-term downregulation of the protein is equivalent to short-term downregulation or if it carries the potential for long-term side effects. To answer this question, long-term observation of the treated patients is mandatory. Certainly closely related is the question of the precise mechanism of action which follows the lower expression of the protein; this should also be explored.

Furthermore, monthly intrathecal injections are not the most convenient ways of application for the patient and our health care system. The increase of dosing intervals or the development of an oral drug would be applauded by many. All these thoughts reflect second steps; the first step has been successfully taken—a therapeutic approach to SOD1-ALS!

## Declarations

**Ethics approval and consent to participate:** Not applicable.

**Consent for publication:** Not applicable.

**Availability of data and materials:** Data related to this study are available from the corresponding author upon reasonable request.
